# Association between geriatric nutritional risk index and pathological phenotypes of IgA nephropathy

**DOI:** 10.7717/peerj.14791

**Published:** 2023-02-13

**Authors:** Yangang Gan, Jiajia Li, Jianping Wu, Rui Zhang, Qianqian Han, Zizhen Li, Qiongqiong Yang

**Affiliations:** Department of Nephrology, Sun Yat Sen Memorial Hospital, Sun Yat-sen University, Guangzhou, China

**Keywords:** IgA nephropathy (IgAN), Geriatric nutritional risk index (GNRI), Serum immunoglobulin G (IgG), Clinical characteristic, Pathological change

## Abstract

**Background:**

IgA nephropathy (IgAN) is an immune disease related to oxidative stress and inflammation. It is the most common type of glomerulonephritis in the world and is the cause of chronic kidney disease and end-stage renal disease (ESRD). The Geriatric Nutritional Risk Index (GNRI) is a practical and uncomplicated method to assess the risk of morbidity and mortality, but its ability to assess IgAN is still unclear. Here, we evaluated the association between the GNRI and clinical and histologic findings of IgAN.

**Methods:**

In a cross–sectional study, we included 348 biopsy-verified IgAN patients. The Oxford classification was used to analyze the pathological characteristics of the included patients. Based on previous studies, the participants were divided into two groups using a cutoff value of 92. Differences in clinicopathological indices between the two groups were compared. The correlation between the GNRI and the indicators was evaluated by using a bivariate correlation analysis. A binary logistic regression analysis was conducted to determine the factors associated with the crescent lesions in IgAN.

**Results:**

In this study, 138 out of 348 patients (39.7%) had low GNRI scores (GNRI < 92). Patients in the low GNRI group tended to have a significantly lower body mass index; lower hemoglobin, serum albumin, serum IgG, and serum C3 levels; and higher 24-h proteinuria. The proportions of females, Oxford M1 and Oxford C1/2 were higher in the low GNRI group. The GNRI was positively correlated with body mass index (r = 0.57, *P* < 0.001), hemoglobin (r = 0.35, *P* < 0.001), serum albumin (r = 0.83, *P* < 0.001), serum IgG (r = 0.32, *P* < 0.001), and serum C3 (r = 0.26, *P* < 0.001) and negatively correlated with 24-h proteinuria (r = −0.36, *P* < 0.001) and the proportion of crescents (r = −0.24, *P* < 0.001). The GNRI scores and serum IgG levels were considered independent factors influencing the crescent lesions in IgAN.

**Conclusions:**

The GNRI can reflect the severity of clinical and histologic phenotypes in IgAN patients. Lower GNRI and serum IgG levels may suggest an increased risk of crescent lesions and are potential markers for disease monitoring in IgAN.

## Introduction

IgA nephropathy (IgAN) is a type of primary glomerulonephritis that has a relatively high incidence worldwide (D’Amico, 1987). IgAN mainly manifests as the deposition of IgA in the glomeruli, a disease that is mediated by immune complexes (Rodrigues, Haas & Reich, 2017). IgA deposition in the mesangial region of the glomerulus causes inflammation that promotes mesangial proliferation and interstitial damage, which progresses to end-stage renal disease (ESRD) in nearly 40% of patients, although some patients maintain stable renal function for many years (Schena et al., 2002). Patients with IgAN have many different clinical manifestations, ranging from asymptomatic hematuria and proteinuria to massive proteinuria to acute renal failure (D’Amico, 2004). Studies have revealed that IgAN may be associated with abnormal activation of the mucosal immune system and chronic inflammation (Novak et al., 2011).

The Geriatric Nutrition Risk Index (GNRI) is an indicator mainly composed of serum albumin levels and body weight, which has the ability to predict the mortality risk of elderly patients with different cancers (Bouillanne et al., 2005; Lidoriki et al., 2021). Several studies on patients with malignant tumors have shown that compared with the use of serum albumin levels, body weight, or body mass index (BMI) alone, the GNRI is advantageous in prognostic evaluation (Kaito et al., 2020; Mizuno et al., 2019). The GNRI can predict recurrence of arrhythmia after catheter ablation of atrial fibrillation (Kaneko et al., 2021). Lower GNRI patients with non-ST-segment elevation acute coronary syndrome have adverse prognosis undergoing percutaneous coronary intervention (Zhao et al., 2020).The GNRI is also a simple tool for clinical prognosis in B-cell lymphoma patients (Chuang et al., 2021). Moreover, one study showed that the GNRI is related to the state of inflammation (Ruan et al., 2021). Currently, renal biopsy is the gold standard for the diagnosis and prognosis of IgAN. Pathological classification combined with clinical factors is widely used in clinical practice to make preliminary predictions regarding the prognosis for IgAN patients. Kidney puncture is an invasive examination, and it is difficult to judge the condition by repeating the examination. Therefore, indicators that predict pathological changes and prognosis need to be explored, particularly noninvasive indices. Given these findings, the GNRI is a practical and simple method to assess the overall condition of patients. However, studies on the association between GNRI and IgAN are lacking; therefore, this study aims to explore the correlation between the GNRI and clinicopathological features of IgAN.

## Methods

### Subjects

This study had a cross-sectional design. Patients who were diagnosed with IgAN *via* renal biopsies from June 2015 to February 2021 were included. The exclusion criteria were as follows: (1) secondary IgAN, such as that associated with systemic lupus erythematosus, diabetic nephropathy, Henoch-Schonlein purpura nephritis, hepatitis B virus-related glomerulonephritis or incomplete data; and (2) acute severe infectious diseases, liver cirrhosis, and cancer. Ultimately, 348 eligible IgAN patients were included. The research protocol was strictly reviewed and approved by the hospital Ethics Committee of Sun Yat-Sen Memorial Hospital, and written informed consent was obtained from all patients before the study (2019-ky-077).

### Clinical and laboratory data

Clinical and laboratory data were collected for all patients. The clinical indicators included sex, age, height, weight, blood pressure, and course of disease. Laboratory data included hemoglobin, serum creatinine, serum albumin, serum cholesterol, serum triglyceride, high-sensitivity C reactive protein (hs-CRP), serum immunoglobulin and complement. The Chronic Kidney Disease Epidemiology Collaboration (CKD-EPI) equation was utilized to calculate the eGFR (Levey et al., 2009). BMI was calculated as weight (kg)/height (m^2^). The GNRI was calculated according to the formula reported in the literature (Ruan et al., 2021). Based on previous research, this study used a GNRI of 92 as the cutoff value (Zheng et al., 2021).

### Renal pathology evaluation

All specimens were evaluated using light microscopy and immunofluorescence. We used the Oxford Classification Scoring System to evaluate cases and classify them according to independent histologic lesions, including mesangial hypercellularity (M), endocapillary hypercellularity (E), segmental glomerulosclerosis (S), interstitial fibrosis/tubular atrophy (T), and cellular/fibrocellular crescents (C) (Trimarchi et al., 2017).

### Statistical analyses

Continuous variables were tested for normality. Normally distributed variables are expressed as the mean ± SD, and non-normally distributed variables are presented as the median and interquartile range. Categorical variables are expressed as frequencies and percentages. A *t* test was used for the comparison of normally distributed continuous variables. The $\chi^2$ test was used to compare the differences between categorical variables. The nonparametric test was also used to compare non-normally distributed variables. A bivariate linear correlation (Pearson or Spearman correlation) analysis was utilized to evaluate the correlation between the GNRI and various factors. A binary logistic regression analysis was conducted to determine the factors associated with the crescent lesions in IgAN. Statistical Product and Service Solutions (SPSS) version 23.0 was utilized for statistical analyses. A *P* $<$ 0.05 was considered statistically significant.

## Results

### Characteristics of patients with IgAN

The characteristics at the time of kidney biopsy were stratified based on the GNRI value and are presented in Table 1. Using a GNRI of 92 as the demarcation point, the patients were divided into two groups, a low GNRI group (GNRI ${\rm \; } < {\rm \; }$92) and a high GNRI group (GNRI $\ge$ 92). The number of patients with a low GNRI score was 138 out of 348 (39.7%). Patients in the low GNRI group were more often female and had a lower BMI, hemoglobin serum albumin, serum IgG and C3. In contrast, the 24-h proteinuria levels of the low GNRI group were higher than those of the high GNRI group. The rate of steroid use was significantly higher in the low GNRI group. The proportion of renin-angiotensin system blocker (RASB) was higher in the high GNRI group. Notably, there were no significant differences in age, SBP, DBP, eGFR, serum creatinine, serum cholesterol, serum triglycerides, hs-CRP, serum IgA, serum IgM, or serum C4 levels between the groups.

**Table 1:** The presentation of data in this study.

Characteristic	GNRI $\; \ge \;$ 92 (*n* = 210)	GNRI $\; < \;$ 92 (*n* = 138)	*P* value
Age (years)	36 (29, 46)	33 (27, 47)	0.47
Men, *n* (%)	100 (47.6)	41 (29.7)	0.001
BMI (kg/m^2^)	23.6 (21.6, 26)	20.9 (19.3, 22.5)	$<$0.001
SBP (mmHg)	123 (115, 137)	124.5 (110, 141.3)	0.60
DBP (mmHg)	81.5 (75, 90.8)	81 (75, 92)	0.74
eGFR (mL/min/1.73 m^2^)	85.6 (62.8, 103)	80.7 (41.3, 108.1)	0.30
24-h proteinuria (g/d)	0.5 (0.2, 1.1)	1.1 (0.4, 2.7)	<0.001
Serum creatinine (μmol/L)	89 (72, 118)	83 (68, 143)	0.82
Serum albumin (g/L)	38.4 (35.5, 40.7)	31 (26.1, 33.6)	$<$0.001
Serum cholesterol (mmol/L)	5 (4.4, 5.7)	5.1 (4.3, 6.2)	0.27
Serum triglycerides (mmol/L)	1.3 (0.9, 1.9)	1.2 (0.9, 1.7)	0.21
Hemoglobin (g/L)	130 (118, 145)	119 (102, 129)	<0.001
Hs-CRP (mg/L)	0.8 (0.3, 2.1)	0.7 (0.3, 2.5)	0.49
Serum IgG (g/L)	12.3 (10.4, 14.3)	10.7 (7.8, 12.7)	$<$0.001
Serum IgA (g/L)	3.4 (2.6, 4.2)	3.2 (2.5, 3.9)	0.10
Serum IgM (g/L)	1.1 (0.8, 1.5)	1.1(0.9, 1.5)	0.39
Serum C3 (mg/L)	1,070 (956, 1,205)	978.5 (865.5, 1,132.5)	$<$0.001
Serum C4 (mg/L)	246 (203, 304.5)	247.5 (204.8, 308.8)	0.64
RASB, *n* (%)	153 (72.9)	84 (60.9)	0.02
Corticosteroid, *n* (%)	45 (21.4)	74 (53.6)	$<$0.001

**Notes:**

Results are presented as median (interquartile range) or number (percent). Groups were compared by using Mann-Whitney U test or chi-square test.

BMI, body mass index; SBP, systolic blood pressure; DBP, diastolic blood pressure; eGFR, estimated glomerular filtration rate; hs-CRP, high sensitivity C reactive protein; RASB, renin-angiotensin system blocker.

### Kidney biopsy results

The pathology results are presented in Table 2. The results revealed that the proportions of M1, C1 and C2 were higher in the low GNRI group than in the high GNRI group (low GNRI *vs* high GNRI: M1, 98.6% *vs* 93.8%, *P* ${\rm \; } = {\rm \; }$0.03; C1, 50.7% *vs* 44.8% and C2, 11.6% *vs* 4.3%, *P* $= {\rm \; }$0.007), whereas the ratios of E1, S1 and T1/2 were not significantly different (low GNRI *vs* high GNRI: E1, 23.9% *vs* 17.6%, *P* ${\rm \; } = {\rm \; }$0.15; S1, 43.5% *vs* 41.9%, *P* $= 0.77;{\rm \; }$T1, 21.7% *vs* 19.1% and T2, 11.6% *vs* 8.1%, *P* $\; = {\rm \; }$0.40) between these two groups.

**Table 2:** Renal pathological injury score comparison in IgAN patients with different nutritional risk.

Renal pathology	Score	GNRI $\; \ge \;$ 92 (*n* = 210)	GNRI $\; < \;$ 92 (*n* = 138)	*P* value
Oxford MEST-C, *n* (%)				
M	M0	13 (6.2)	2 (1.5)	
	M1	197 (93.8)	136 (98.6)	0.03
E	E0	173 (82.4)	105 (76.1)	
	E1	37 (17.6)	33 (23.9)	0.15
S	S0	122 (58.1)	78 (56.5)	
	S1	88 (41.9)	60 (43.5)	0.77
T	T0	153 (72.9)	92 (66.7)	
	T1	40 (19.1)	30 (21.7)	
	T2	17 (8.1)	16 (11.6)	0.40
C	C0	107 (51)	52 (37.7)	
	C1	94 (44.8)	70 (50.7)	
	C2	9 (4.3)	16 (11.6)	0.007

**Notes:**

Categorical variables were expressed as number (percent).

MEST-C, M, mesangial hypercellularity; E, endocapillary hypercellularity; S, segmental glomerulosclerosis; T, interstitial fibrosis/tubular atrophy; C, crescents formation.

### Characteristics of IgAN patients with crescents

Of the 348 IgAN patients, 25 (7.2%) and 164 (47.1%) patients were assigned to C2 and C1, respectively. The remaining 159 patients (45.7%) were classified as C0. The results are shown in Table 3. The C ≥ 1 group was characterized by a significantly lower BMI, GNRI, serum albumin and serum IgG and a higher 24-h proteinuria, serum cholesterol and a higher rate of corticosteroid use. There were no significant differences in age, sex, SBP, DBP, eGFR, hemoglobin, serum triglycerides, serum creatinine, Hs-CRP, serum IgA, serum IgM, serum complement levels or the rate of RASB use between the groups.

**Table 3:** Baseline clinicopathological characteristics of IgAN patients with crescents.

Variables	C0 (*n* $=$ 159)	C1/2 (*n* $= 189$)	*P* value
Age (years)	36 (29, 48)	34 (28, 45)	0.19
Men, *n* (%)	67 (42.1)	74 (39.2)	0.57
BMI (kg/m^2^)	22.9 (21, 25.3)	22 (20.2, 24.3)	0.02
SBP (mmHg)	123 (113, 137)	124.5 (114.3, 138)	0.83
DBP (mmHg)	81 (74, 90.5)	81 (75.3, 91)	0.57
eGFR (mL/min/1.73 m^2^)	87.4 (51.7, 107.1)	81.8 (57.6, 101.6)	0.59
24-h proteinuria (g/d)	0.4 (0.2, 1.5)	0.7 (0.4, 1.8)	0.001
Serum creatinine (μmol/L)	83 (69.5, 123.5)	90.5 (71, 122.8)	0.50
Serum albumin (g/L)	36.3 (33.7, 39.9)	34.3 (30.9, 38.4)	0.001
Serum cholesterol (mmol/L)	4.9 (4.2, 5.8)	5.1 (4.5, 6.1)	0.03
Serum triglycerides (mmol/L)	1.3 (0.9, 1.9)	1.3 (0.9, 1.7)	0.79
Hemoglobin (g/L)	126.7 ± 20.6	123.8 ± 21.5	0.20
Hs-CRP (mg/L)	0.8 (0.3, 2.7)	0.7 (0.3, 2.2)	0.96
GNRI	97.6 (90.2, 104.6)	93.1 (85.4, 101)	0.001
Serum IgG (g/L)	12.4 (10.3, 14.4)	11 (9, 12.9)	$<$0.001
Serum IgA (g/L)	3.3 (2.5, 4.1)	3.2 (2.6, 4)	0.94
Serum IgM (g/L)	1.1 (0.8, 1.5)	1.1 (0.8, 1.5)	0.58
Serum C3 (mg/L)	1,040 (900, 1,195)	1,030 (933.5, 1,170)	0.62
Serum C4 (mg/L)	246 (194, 307)	246 (205.5, 306.5)	0.59
RASB, *n* (%)	101 (63.5)	136 (72)	0.09
Corticosteroid, *n* (%)	37 (23.3)	82 (43.4)	$<$0.001

**Notes:**

Results are presented as the mean ± SD, median (interquartile range) or number (percent). Groups were compared by using *t*-test, Mann-Whitney U test or chi-square test.

BMI, body mass index; SBP, systolic blood pressure; DBP, diastolic blood pressure; eGFR, estimated glomerular filtration rate; hs-CRP, high sensitivity C reactive protein; GNRI, geriatric nutritional risk index; RASB, renin-angiotensin system blocker.

### Relationship between GNRI and clinicopathological parameters

**Figure 1:**
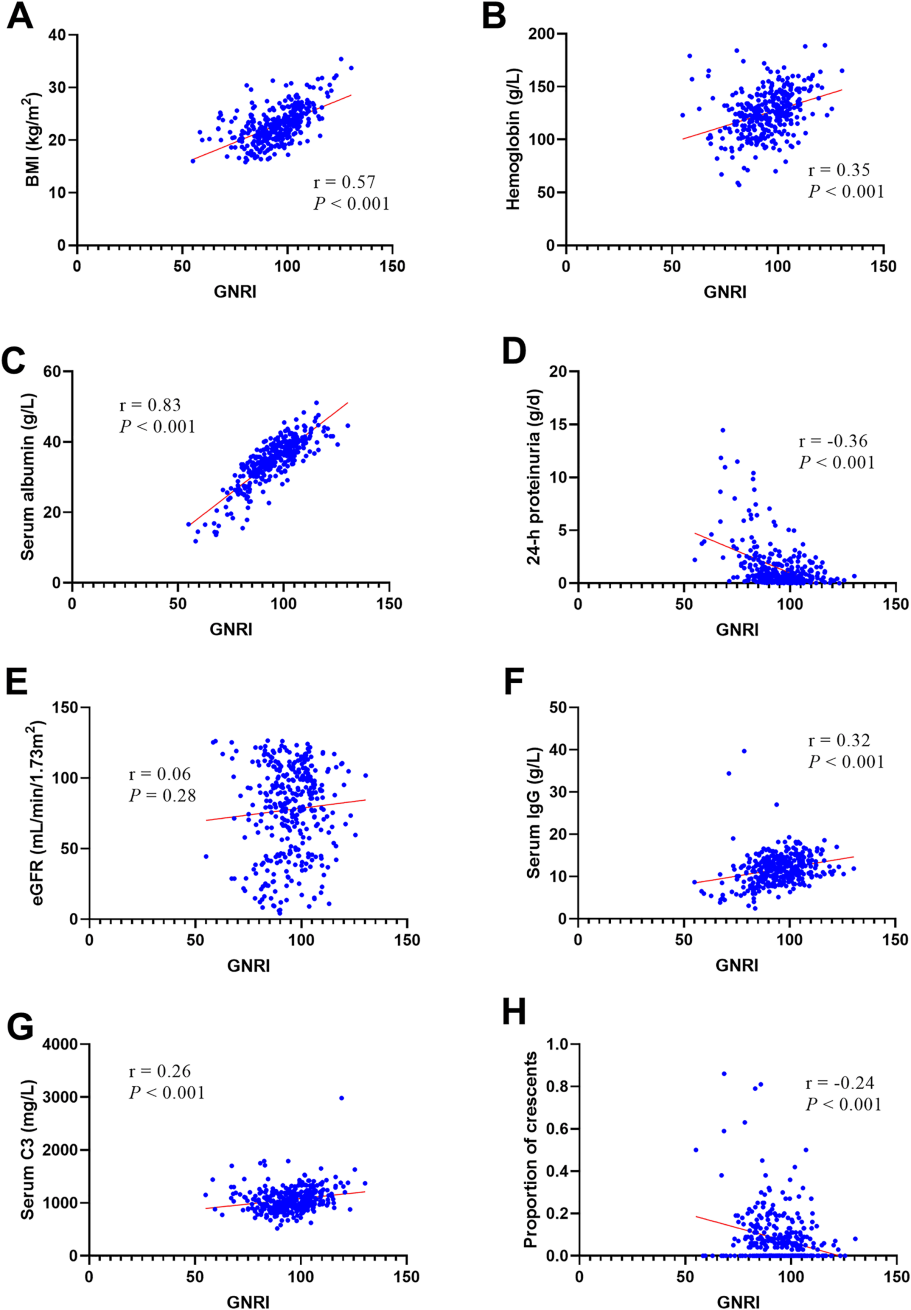
The Person’s or Spearman’s analysis of GNRI and related factors. BMI (A), Hemoglobin (B), serum albumin (C), 24-h proteinuria (D), eGFR (E), serum IgG (F), serum C3 (G), proportion of crescents (H). Abbreviations: BMI, body mass index; eGFR, estimated glomerular filtration rate.

According to the bivariate linear correlation analysis presented in Table 4 and Fig. 1, the GNRI was negatively correlated with 24-h proteinuria (r $=$ 0.36, *P* $<$ 0.001) and the proportion of crescents (r $=$ 0.24, *P* $<$ 0.001) and positively correlated with BMI (r $\; = 0.57$, *P* $<$ 0.001), hemoglobin (r $\; = 0.35$, *P* $<$ 0.001), serum albumin (r $\; = 0.83$, *P* $<$0.001), serum IgG (r $\; = 0.32$, *P* $<$ 0.001), and serum C3 (r $\; = 0.26$, *P* $<$ 0.001). There was no statistically significant difference in the GNRI with eGFR.

**Table 4:** Analysis of the factors associated with GNRI in IgAN.

Indexes	Correlation coefficient	95% CI		*P* value
		Lower	Upper	
BMI	0.57	0.48	0.64	$<$0.001
Hemoglobin	0.35	0.22	0.47	$<$0.001
Serum albumin	0.83	0.78	0.86	$<$0.001
24-h proteinuria	−0.36	−0.46	−0.26	$<$0.001
eGFR	0.06	−0.05	0.17	0.28
Serum IgG	0.32	0.21	0.42	$<$0.001
Serum C3	0.26	0.15	0.36	$<$0.001
Proportion of crescents	−0.24	−0.34	−0.14	$<$0.001

**Note:**

BMI, body mass index; eGFR, estimated glomerular filtration rate.

### Analysis of the influencing factors associated with thecrescent lesions

The correlative factors for crescent lesions in patients with IgAN were determined using a logistic regression, as shown in Table 5. Given that the GNRI is colinear with BMI and serum albumin, the GNRI is composed of these two parts. Finally, serum cholesterol, serum IgG, GNRI and 24-h proteinuria were included for the analyses. After adjustment for serum cholesterol and 24-h proteinuria, the independent influencing factors were GNRI (OR 0.98, 95% CI [0.96–0.99]) and serum IgG (OR 0.90, 95% CI [0.84–0.97]).

**Table 5:** Logistic regression for the influencing factors associated with crescent lesions.

Variables	B	SE	Wald $\chi^2$	OR (95% CI)	*P* value
Serum cholesterol	−0.04	0.08	0.27	0.96 [0.83–1.11]	0.60
Serum IgG	−0.10	0.04	8.27	0.90 [0.84–0.97]	0.004
GNRI	−0.02	0.01	4.67	0.98 [0.96–0.99]	0.03
24-h proteinuria	−0.06	0.07	0.90	0.94 [0.82–1.07]	0.34

**Note:**

GNRI, geriatric nutritional risk index.

## Discussion

In the present study, we identified a correlation between the GNRI and the clinicopathology in IgAN patients. A total of 348 patients with IgAN were included in the analysis. The results showed that the prevalence of high nutritional risk was 39.7%, based on the cutoff value of 92. IgAN patients with decreased GNRI exhibited a severe clinicopathologic condition. These findings may indicate that the GNRI can be used to screen the status of IgAN patients.

The GNRI has been used as an indicator to reflect the systemic inflammatory response (Ruan et al., 2021) and nutritional state (Lin & Hung, 2019). Previous studies have shown the value of the GNRI score in the prognosis of patients with malignant tumors. Yamana et al. (2015) found that the GNRI can reflect nutritional status and the incidence of complications in esophageal cancer patients. Moreover, patients with CKD and lower GNRI scores have an increased risk of progression to ESRD, cardiovascular events and mortality (Lin & Hung, 2019; Xiong et al., 2020). The components of the GNRI are mainly serum albumin levels, height and body weight. With regard to weight loss, the GNRI mainly reflects the conditions of systematic disease and protein-calorie malnutrition that are observed in patients with acute stress (Gu et al., 2015). In the GNRI formula, serum albumin has much more influence than body weight (Bouillanne et al., 2005). In our study, GNRI was more strongly correlated with serum albumin than BMI. Serum albumin is an indicator of inflammation of the microenvironment (Evans, 2021). High levels of various inflammatory cytokines and oxidative stress have been found in patients with IgAN (Apeland et al., 2020). Furthermore, Kawai et al. (2018) found that low levels of serum albumin are an independent risk factor for ESRD in IgAN. The increased cytokines of inflammation decrease the synthesis of albumin and its mRNA in the liver during inflammation (Huang et al., 1995). The inflammatory factor interleukin-2 can cause albumin loss, and oxidative stress can cause albumin degeneration (Bito et al., 2005). Proteinuria associated with severe clinical and histologic findings in IgAN and is also an independent predictor of progression in IgAN (Zhao et al., 2016). We also found that the GNRI was negatively associated with 24-h proteinuria. Increased proteinuria may also accelerate to hypoalbuminemia. Indeed, proteinuria also reflects systemic inflammation and endothelial dysfunction (Paisley et al., 2003). Overall, the GNRI, which is composed of serum albumin and body weight, may have the ability to improve the prediction of IgAN status. However, no studies have attempted to apply the GNRI to evaluate the status of IgAN patients.

The prevalence of high nutritional risk was higher in female patients than in male patients in our study. Therefore, clinicians should focus on female patients, as the rate of high nutritional risk differs by sex. Wang et al. (2019) found that renal anemia in IgAN is associated with sex, hypoproteinemia, reduced eGFR levels and severe renal tubulointerstitial lesions. This may reflect the sex difference in the severity of anemia in IgAN, as female patients had more serious symptoms. In this study, we demonstrated that the GNRI is positively correlated with hemoglobin. Because GNRI can reflect the status of nutrition and inflammation, this may impact the synthesis of hemoglobin. Serum creatinine, eGFR, proteinuria and the Oxford classification are generally used for the diagnosis and classification of IgAN (Hao et al., 2019). This study did not find that the GNRI scores were related to serum creatinine and eGFR as calculated, but they were related to histological lesions. This suggests that serum creatinine and eGFR cannot fully reflect renal lesions, particularly for those with low muscle mass. Research has shown that the function of the kidney can be overestimated due to poor nutritional status and reduced muscle mass (Perrone, Madias & Levey, 1992; Yoo et al., 2019). Patients with reduced muscle metabolism may have decreased production of serum creatinine. In addition, emerging indicators are better able to predict the prognosis of patients with IgAN. Increased levels of serum IgG are associated with a higher rate of cumulative renal survival (Liu et al., 2019). A reduced serum IgG could be attributable to an increased urinary IgG extraction in IgAN patients with increased proteinuria (Widstam-Attorps & Berg, 1991; Tang et al., 2020). Research suggests that a decrease in serum IgG levels at renal biopsy was independently associated with an increased risk of a poor kidney outcome in IgAN (Liu et al., 2019). In our study, serum IgG was found to be associated with crescents in patients with IgAN, which may partly explain the poor prognosis of patients with a reduced serum IgG.

Complement activation induced by the pathogenic immune complexes of galactose-deficient IgA1 is involved in the progression of IgAN. The loss of renal function correlates with activated C3 and mesangial C3 deposition (Chen et al., 2019). Although partial local glomerular complement activation occurs, most serum complement levels remain within the normal range (Mizerska-Wasiak et al., 2015). Kim et al. (2012) found that low serum C3 levels and mesangial C3 deposition were independent risk factors for progression of renal in IgAN. What’s more, many studies have found that the IgA/C3 ratio could reflects the severity of disease and the progression of nephropathy in IgAN patients (Mizerska-Wasiak et al., 2015; Komatsu et al., 2004; Zhang et al., 2013). However, no difference was seen between IgA/C3 ratios in different crescent lesions (Mizerska-Wasiak et al., 2015). In our study, GNRI mainly associated with the crescent lesions of IgAN, which reveals GNRI and IgA/C3 ratios could play complementary roles in evaluating the severity of IgAN. Increased complement activation, inflammatory state, and urinary protein excretion in IgAN patients can lead to C3 consumption. Indeed, serum C3 levels are positively correlated with metabolic markers such as BMI, serum albumin, and hemoglobin (Ohsawa et al., 2010). We also found that the GNRI was positively associated with serum IgG and C3 levels, which may indicate that low GNRI levels may reflect immune complex formation and complement activation.

IgAN is a group of diseases that mainly manifest as glomerular mesangial cell proliferation and immune complex deposition (A Working Group of the International IgA Nephropathy Network and the Renal Pathology Society et al., 2009). IgA immune complexes with or without IgG and C3 deposit in the glomerular mesangial area (Novak et al., 2001). We found that mesangial proliferation (M1) and crescent lesion scores (C1/2) were higher in the low GNRI group. These findings suggested that the GNRI can reflect renal pathological injury, particularly for Oxford M and C. One report found that C3 deposition in mesangial cells is related to Oxford M, which is also a risk factor for kidney outcome in children with IgAN (Wu et al., 2021). In the present study, we also found that patients in the low GNRI group had lower serum C3 levels and an increased Oxford M1 lesions. This may be due to more severe complement activation in patients with a low GNRI, which promotes the Oxford M1 lesions. (Haas et al., 2017) found that the presence of any cellular or fibrocellular crescents had a negative effect on renal outcome in IgAN patients, independent of clinical and histological parameters, including proteinuria, eGFR at biopsy, mean arterial pressure and Oxford MEST scores. Generally, Oxford C1 indicates that patients may have poorer kidney outcomes compared with Oxford C0. Indeed, Oxford C2 can identify patients at high risk of progression, whether or not they receive immunosuppressive therapy (Haas et al., 2017). Notably, the crescents can be partially reversed. Therefore, exploring related factors and assessing status in a timely manner have great clinical value for improving the prognosis of IgAN patients with crescent lesions. In our study, GNRI scores were associated with the proportion of crescents. We also found that the GNRI score and serum IgG levels were independent factors influencing the prevalence of crescent lesions in IgAN. These findings indicate that GNRI and serum IgG levels may be potential markers for monitoring crescent formation in IgAN patients. In the future, we need to further explore whether increasing GNRI levels can reverse crescentic lesions.

### Study limitations

There are some limitations in this study. This study may have limitations of a cross-sectional study. Indeed, our information was only from kidney biopsy patients, and some patients did not initially undergo renal biopsy for various reasons. Therefore, missing data and selection bias may be present in our study.

## Conclusions

We found that lower GNRI scores were associated with severe clinical and histologic phenotypes in patients with IgAN. Moreover, the low GNRI and serum IgG levels may suggest an increased risk of crescent lesions and are potential markers for disease monitoring in IgAN. Further studies are needed to explore the value of the GNRI in predicting the prognosis of patients with IgAN.

## Supplemental Information

10.7717/peerj.14791/supp-1 Supplemental Information 1 Study data.

10.7717/peerj.14791/supp-2 Supplemental Information 2 Codebook.

## Additional Information and Declarations

### Data Availability

The following information was supplied regarding data availability: The raw measurements are available in the Supplemental Files.
