# Supplementary material for: Association between geriatric nutritional risk index and pathological phenotypes of IgA nephropathy
Source: PeerJ. 2023 Feb 13;11:e14791. doi: 10.7717/peerj.14791 (PMC9933742; doi:10.7717/peerj.14791)
Supplement: Supplemental Information 2 [file peerj-11-14791-s002.docx]

MEST-C, M, mesangial hypercellularity (M0, M1); E, endocapillary hypercellularity (E0, E1); S, segmental glomerulosclerosis (S0, S1); T, interstitial fibrosis/tubular atrophy (T0, T1, T2); C, crescents formation (C0, C1, C2).

SEX: Male and Female

RASB: yes = receiving RASB treatment

Corticosteroid: yes = receiving corticosteroid treatment
